# Monolithically‐Integrated van der Waals Synaptic Memory via Bulk Nano‐Crystallization

**DOI:** 10.1002/advs.202510961

**Published:** 2025-08-26

**Authors:** Jinhyoung Lee, Gunhyoung Kim, Hyunho Seok, Sujeong Han, Hyunwoo Shim, Yoonmi Cha, Sihoon Son, Hyunbin Choi, Magdalena Grzeszczyk, Aleksander Bogucki, Yunseok Choi, Seungil Kim, Hyeonjeong Lee, Chaerin Park, Geonwook Kim, Hosin Hwang, Hyunho Kim, Dongho Lee, Seowoo Son, Geumji Back, Hyelim Shin, Donghwan Choi, Alexina Ollier, Yeon‐Ji Kim, Lei Fang, Gyuho Han, Goo‐Eun Jung, Youngi Lee, Hyeong‐U Kim, Kenji Watanabe, Takashi Taniguchi, Sanghoon Bae, Andreas Heinrich, Won‐Jun Jang, Taesung Kim

**Affiliations:** ^1^ School of Mechanical Engineering Sungkyunkwan University (SKKU) Suwon‐si Gyeonggi‐do 16419 South Korea; ^2^ Center for Quantum Nanoscience Institute for Basic Science (IBS) Seoul 03760 South Korea; ^3^ Department of Semiconductor Convergence Engineering Sungkyunkwan University Suwon 16419 South Korea; ^4^ Research Laboratory of Electronics Massachusetts Institute of Technology Cambridge MA 02139 USA; ^5^ Park Systems Corporation 109, Gwanggyo‐ro, Yeongtong‐gu Suwon‐si Gyeonggi‐do 16229 South Korea; ^6^ SKKU Advanced Institute of Nanotechnology (SAINT) Sungkyunkwan University Suwon 16419 South Korea; ^7^ Department of Nano Science and Technology Sungkyunkwan University Suwon 16419 South Korea; ^8^ Department of Physics Ewha Womans University Seoul 03760 South Korea; ^9^ Department of Mechanical Engineering and Materials Science and Institute of Materials Science and Engineering Washington University in St. Louis Missouri MO 63130 USA; ^10^ Semiconductor Manufacturing Research Center Korea Institute of Machinery and Materials (KIMM) Daejeon 34103 South Korea; ^11^ Nano‐Mechatronics KIMM Campus University of Science & Technology (UST) Daejeon 34113 South Korea; ^12^ National Institute for Materials Science Namiki 1‐1 Tsukuba Ibaraki 305‐0044 Japan; ^13^ Department of Nano Engineering Sungkyunkwan University Suwon 16419 South Korea

**Keywords:** 1S1R cell, 2D/3D heterostructures, atomic force microscopy, resistive switching, synaptic memory

## Abstract

Owing to the evolution of data‐driven technologies, including the large language models, generative artificial intelligence, autonomous driving, and the internet of things requires advanced memory technology. However, conventional memory device structures and fabrication process have significant limitations for high‐density integration. Herein, this study reports the monolithically‐integrated 1‐selector and 1‐resistive (1S1R) synaptic memory in van der Waals (vdW) heterostructure, which overcomes the conventional limitations of device integration technologies. Single‐step direct synthesis of vdW heterostructure and its corresponding 1S1R cell is fabricated via plasma‐enhanced lattice‐distortion. Scanning‐transmission electron microscopy, and X‐ray photoelectron spectroscopy are correlatively applied to observe the effects of plasma‐enhanced nano‐crystallization of bulk vdW VSe_2_. Furthermore, bipolar resistive switching dynamics have been spatially resolved with conductive atomic force microscopy. Furthermore, the artificial vdW heterostructure exhibits the synaptic functionality with interfacial charge accumulation at the 2D/3D interface, enabling linear weight updates across multiple resistance states with minimal nonlinearity. In conclusion, it envision that the monolithically‐integrated 1S1R cell can offers a systematic device platform for next‐generation vdW electronics and its corresponding monolithic 3D integration.

## Graphical abstract

Monolithically‐integrated van der Waals synaptic memory is presented via bulk nano‐crystallization, which overcomes the conventional limitations of 3D device integration technologies. Furthermore, bipolar resistive switching (LRS/HRS) dynamics is spatially resolved with conductive atomic force microscopy, scanning‐transmission electron microscopy, and X‐ray photoelectron spectroscopy. The monolithic‐integrated 1S1R device exhibits the low leakage currents, robust switching ratios, and reliable bipolar memory states with synaptic response. The monolithically‐integrated resistive memory will offer the generalizable platform for next‐generation 3D integrated neuromorphic device and edge‐computing AI hardware.

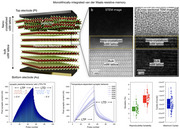

## 1 Introduction

In the era of ubiquitous computing and artificial intelligence (AI), the ever‐increasing demand for real‐time, on‐site data processing necessitates the development of neuromorphic hardware^[^ ^1^ ^]^ capable of integrating memory and computing functionalities within a single unit. Neuro‐inspired devices, particularly memristors, have garnered considerable attention as promising candidates for edge‐computing applications due to their inherent capability to emulate synaptic behaviors with high energy efficiency^[^ ^2^ ^]^ and device density.^[^ ^3^ ^]^ Operating through the modulation of ion migration^[^ ^4^ ^]^ and defect dynamics, memristors enable multilevel, non‐volatile resistance switching, thereby offering a viable platform for in‐memory computing^[^ ^5^ ^]^ architectures. Recent advancements in AI and data‐centric technologies, including large language models, autonomous vehicles, and the Internet of Things (IoT), have further accelerated the demand for next‐generation memory systems with enhanced scalability, endurance, and integration capability. While conventional charge‐based memory technologies have benefitted from CMOS scaling,^[^ ^6^ ^]^ their device architectures face intrinsic limitations in terms of power consumption and integration density.

Hence, resistive random‐access memory (ReRAM), particularly those based on a metal–insulator–metal (MIM) configuration, has emerged as a compelling alternative owing to its structural simplicity and compatibility with crossbar architectures. Despite the promise of ReRAM, most polycrystalline metal oxide‐based memristive devices, such as those utilizing TiOx,^[^ ^7^ ^]^ TaOx,^[^ ^8^ ^]^ or HfOx,^[^ ^9^ ^]^ exhibit significant leakage currents through grain boundaries, which result in poor off‐state behavior and low switching reliability. While the use of amorphous active layers^[^ ^10^ ^]^ mitigates leakage by eliminating grain boundary^[^ ^11^ ^]^ pathways, it introduces new challenges in precisely controlling the formation and dissolution of conductive filaments, thereby limiting device uniformity and long‐term reliability.^[^ ^12^ ^]^ Strategies such as dislocation‐guided filament confinement^[^ ^13^ ^]^ in single‐crystalline SiGe or field‐enhanced conical electrode design have been introduced to improve reproducibility^[^ ^14^ ^]^; However, the integration of selector devices is essential to mitigate sneak‐path currents in crossbar array architectures, which is a critical prerequisite for enabling the reliable and scalable implementation of resistive memory in very‐large‐scale integration (VLSI) systems.

To address the scalability and integration challenges of planar architectures, the semiconductor industry has increasingly focused on three‐dimensional heterogeneous integration (3DHI),^[^ ^15^ ^]^ wherein disparate functional layers, such as memory, logic, and optoelectronics, are vertically stacked to form compact, multifunctional systems. While 3DHI offers significant benefits in performance and footprint, its reliance on through‐silicon vias and wafer bonding techniques introduces formidable fabrication complexities and alignment challenges. As an alternative, monolithic 3D integration (M3D),^[^ ^16^ ^]^ wherein functional device layers are sequentially fabricated and integrated without individual wafer bonding, holds promise for achieving seamless vertical integration.^[^ ^17^ ^]^ However, the mechanical fragility^[^ ^18^ ^]^ and intrinsic stress^[^ ^19^ ^]^ of conventional materials pose serious barriers to the practical realization of M3D integration, especially during substrate detachment and layer transfer processes.^[^ ^20^ ^]^ In contrast, two‐dimensional (2D) van der Waals (vdW) materials^[^ ^3^, ^21^ ^]^ provide a transformative opportunity to overcome these constraints. Their atomically thin nature, exceptional mechanical flexibility,^[^ ^22^ ^]^ and negligible internal stress^[^ ^23^ ^]^ make them ideally suited for M3D integration.^[^ ^24^ ^]^ Moreover, vdW materials maintain electrical performance^[^ ^25^ ^]^ comparable to bulk silicon‐based devices,^[^ ^26^ ^]^ thereby offering a compelling platform for high‐density,^[^ ^27^ ^]^ low‐power memory^[^ ^28^ ^]^ and logic applications.^[^ ^29^ ^]^ Nevertheless, several limitations have limited the commercial adoption of vdW M3D integration. These include i) the difficulty of achieving precise control over vdW stacking kinetics, ii) the accumulation of polymeric residues and mechanical warpage at vdW interfaces, and iii) the large‐area scalability of vdW crystallinity.

Herein, we report the monolithically‐integrated 1S1R cell in vdW 2D/3D heterostructure, offering the significant breakthrough of conventional vdW integration technologies. To achieve the monolithically‐integrated 1S1R cell, vdW nano‐crystallization has been conducted with Ar + H_2_S plasma sulfurization, inducing the penning effects and ion penetration. Direct synthesis of vdW 2D/3D heterostructure and its corresponding 1S1R synaptic device performance were clearly demonstrated, which has not been possible previously. Unlike heterogeneous stacks, this artificial vdW 2D/3D heterostructure can be fabricated without additional selector materials or complex 3D stacking, which eliminates the interface mismatches and parasitic leakage. The consistent yield and uniform switching behavior observed across 50 devices further demonstrate the scalability and reliability of the monolithic 1S1R architecture. Moreover, bipolar resistive switching dynamics has been spatially resolved with conductive atomic force microscopy (C‐AFM) with the *I*_max_, *I*_min_, and *I*_HRS_ have been measured as 6.91 × 10^−10^ A, 1.30 × 10^−13^ A, and 1.27 × 10^−10^ A. Based on these current values, selectivity (*I*_max_/*I*_HRS_) and on/off ratio (*I*_max_/*I*_min_) can be calculated as 5.44 × 10^0^ and 5.61 × 10^3^. HRS (high resistance state)/LRS (low resistance state) current value statically measured as 0.137 nA in HRS [state “0”], 0.851 nA in LRS [state “1”], and 0.132 nA in HRS [state “0”]. Moreover, plasma sulfurization is processed within a top‐down approach, vdW 2D/3D heterostructure can be reliably fabricated regardless of vdW stacking order, vdW layer numbers, and vdW lattice type. Regarding this systematic expandability, we envision that our monolithically‐integrated 1S1R cell can offers a systematic platform for next‐generation M3D integration and advanced vdW integration.

## 2 Results and Discussion

### 2.1 Monolithically‐Integrated 1S1R Cell in vdW 2D/3D Heterostructure

**Figure 1:**
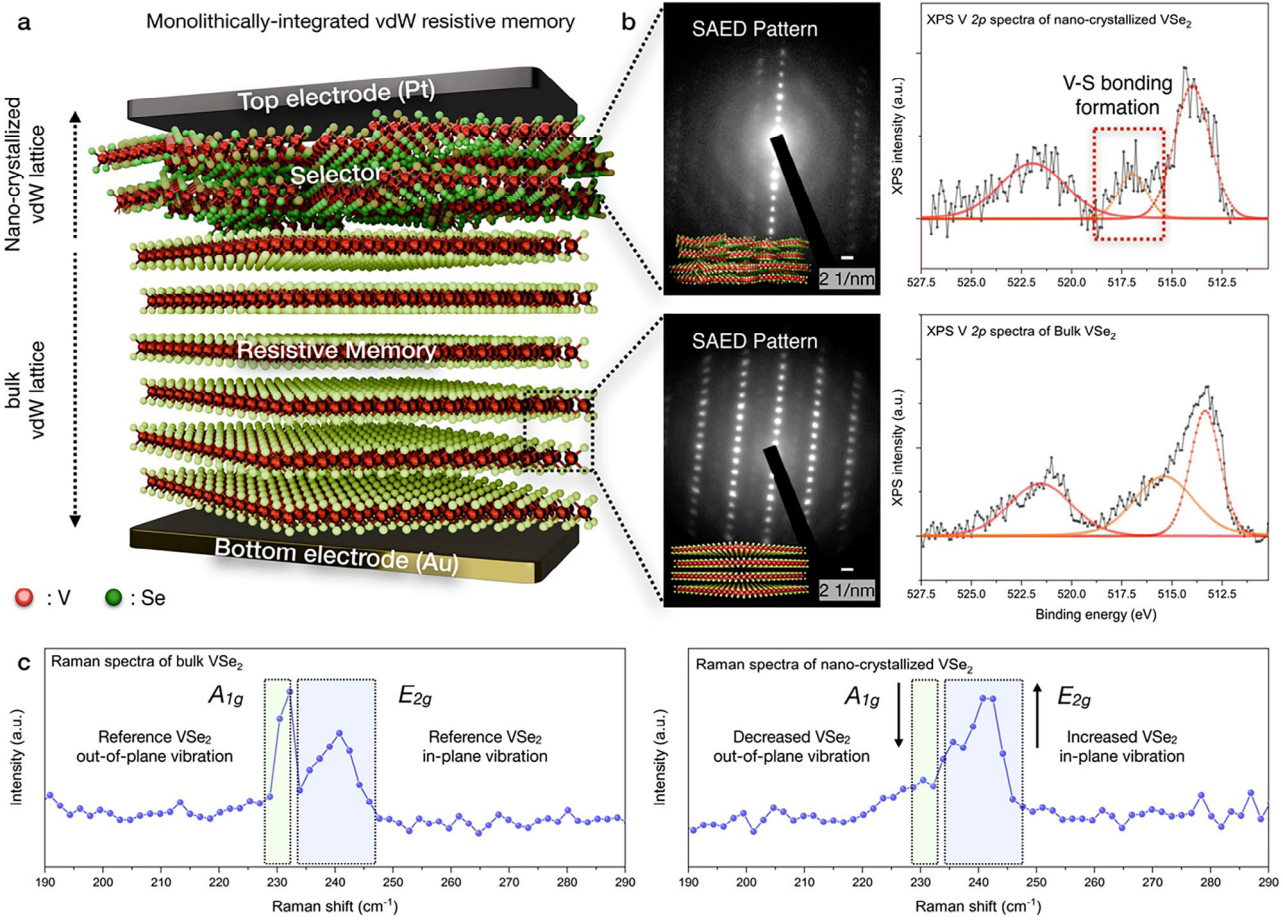
Device configuration of monolithically‐integrated vdW synaptic memory. a) Schematic illustration of monolithically‐integrated vdW synaptic memory, which is constructed with nano‐crystallized vdW VSe_2_ lattice (selector) and bulk vdW VSe_2_ lattice (resistive memory). Electrodes for resistive switching were selected as Platinum (top) and gold (bottom). b) SAED patterns and XPS V *2p* spectra of bulk VSe_2_ and nano‐crystallized VSe_2_, resulting the nano‐crystallization and V─S bonding formation during the Ar + H_2_S plasma treatment. c) Raman spectra of bulk VSe_2_ and nano‐crystallized VSe_2_, indicating the decreased *A*_1g_ peak and increased *E*_2g_ peak.

To monolithically integrate the vdW 2D/3D heterostructure, vdW VSe_2_ has been nano‐crystallized with Ar + H_2_S plasma‐enhanced lattice‐distortion techniques. As shown in **Figure** **1**a, Schematic illustration of monolithically‐integrated vdW synaptic memory, which is constructed with nano‐crystallized vdW VSe_2_ (selector, 4.49 nm) and bulk vdW VSe_2_ (resistive memory, 37.3 nm). As nano‐crystallized vdW VSe_2_ and bulk vdW VSe_2_ operates as switching medium of conductive filaments, electrodes for resistive switching were selected as platinum (top electrode) and gold (bottom electrode). Selected area electron diffraction (SAED) patterns and XPS V *2p* spectra of bulk VSe_2_ and nano‐crystallized VSe_2_ directly indicating the nano‐crystallization and V─S bonding formation during the Ar + H_2_S plasma treatment (Figure 1b). As our previous research revealed the number of vdW layer of the nano‐crystallized vdW materials (Bi_2_Se_3_,^[^ ^30^ ^]^ VSe_2_^[^ ^31^ ^]^) can be precisely controlled with RF plasma power variation. While the ratio between nano‐crystallization (selector) and bulk vdW materials (resistive memory) can be modulated with RF plasma power variation, 1S1R cell functionality can be artificial modulated with RF plasma power variation, enabling the potential applications of wafer‐scale M3D integration of vdW electronics. Also, Raman spectroscopy of nano‐crystallized vdW VSe_2_ and bulk vdW VSe_2_ has been conducted in Figure 1c. Owing to the nano‐crystallization, *A*_1g_ peak has been decreased, while *E*_2g_ peak was reversibly increased. Such Raman peak redistribution corresponds to the structural reconstruction of nano‐crystallized VSe_2_, validating the grain boundary (decreased *E*_2g_ peak) and sulfur intercalation (increased *A*_1g_ peak) (Figure S1, Supporting Information). In contrast to previous 1S1R architectures that rely on multi‐step heterogeneous stacking and high‐temperature growth, our artificial vdW 2D/3D heterostructure is synthesized via a single‐step plasma sulfurization (≈5 min) at room temperature over >cm^2^ scale. This approach eliminates additional selector deposition and complex 3D stacking, thereby reducing fabrication steps by more than 50% compared with recent reports,^[^ ^32^ ^]^ while ensuring high device yield and uniform switching behavior across 50 devices. The combination of single‐step, low‐temperature, and wafer‐scale fabrication directly addresses the scalability challenges of conventional 3D integration.

### 2.2 Atomic‐Scale Observation of Monolithically‐Integrated vdW 2D/3D Heterostructure

**Figure 2:**
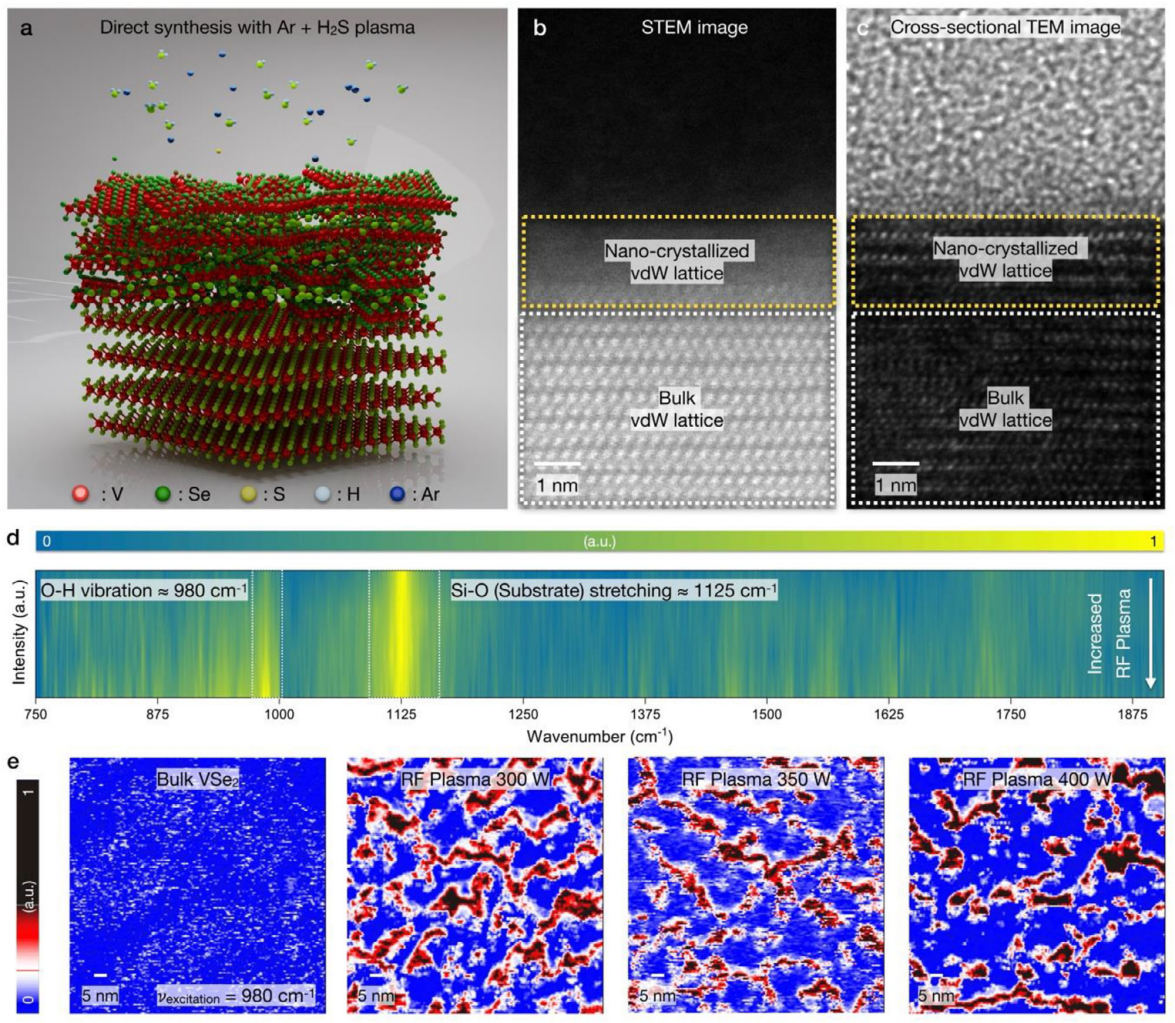
Atomic‐scale observation of nano‐crystallized VSe_2_. a) Schematic illustration of nano‐crystallization of VSe_2_, indicating the ion‐penning effects and ion penetration effects of bulk vdW VSe_2_. Cross‐sectional b) STEM and c) TEM image of nano‐crystallized VSe_2_, enabling the monolithically‐integrated vdW resistive memory fabrication. d) A sequential cascade of IR spectra, correlating the increased O‐H stretching peak (980 cm^−1^) with RF plasma power. e) Spatially‐resolved GB passivation with PiFM imaging (excitation wavenumber: 980 cm^−1^). During the Ar + H_2_S plasma treatment, the H atom and native oxide chemically bond with an exposed metal atom, directly O─H bonding formation corresponds to the GB passivation effects.

During the Ar + H_2_S plasma treatment, the penning effects and ion penetration have been activated (**Figure** **2**a). H_2_S^+^ generation directly derives the ion Penning effect by the Ar gas and the direct ionization of H_2_S as followed Equations (1) and (2).^[^ ^33^ ^]^

(1) $$\mathrm{Ar}+e^{-}\to Ar^{+}+2e^{-}$$

(2) $$Ar^{+}+H_{2}S\to \mathrm{Ar}+H_{2}S^{+}$$

Sufficient electrons in the plasma system can directly ionize H_2_S gas to generate H_2_S^+^, as shown in Equation (3). But except Ar gas, it is difficult to generate H_2_S plasma despite the low ionization energy of H_2_S gas.^[^ ^34^ ^]^

(3) $$e^{-}+H_{2}S^{+}\to 2e^{-}$$

For the precise control of plasma‐based sulfurization, gas mixture ratio was settled as Ar gas and H_2_S gas as 1:1 ratio (50 sccm injection for each gas). Regarding our previous articles, RF power variation can control the penetration vdW layer. Nano‐crystallization effect has been observed with cross‐sectional STEM imaging (Figure S2, Supporting Information) and energy‐dispersive X‐ray spectroscopy (EDS) mapping (Figure S3, Supporting Information) in Figure 2b,c, exhibiting the nano‐grain (lateral dimension as ≈5 nm) formation by lattice‐distortion under E‐field driven ion bombardment. After plasma treatment, vdW interface has been remained clean vdW interface unlike conventional vdW integration method. To laterally observe the nano‐grain distribution and RF plasma power dependency, nano‐crystallized VSe_2_ was sequentially analyzed within PiFM measurements. Owing to hydroxy group adsorption at the nano‐grain, PiFM measurement can spatially detect the O‐H stretching peak, directly guiding the spatial nano‐grain distribution. Additionally, cascade of infrared (IR) spectra with sub‐10 nm resolution directly supports the dominance of 980 cm^−1^ peak within increased RF plasma power (Figure 2d). As PiFM spatially resolves chemical composition with sub‐10 nm resolution,^[^ ^35^ ^]^ pristine VSe_2_ indicates absence of photo‐induced force signal. Increasing the RF plasma power to 300 W, spatial heterogeneity has been observed with excitation wavenumber 980 cm^−1^. Spatial heterogeneity of photo‐induced force was dominantly derived from nano‐crystallization. When nano‐crystallization has been generated, metal atoms are exposed to chemisorb with hydrogen atoms. At the ambient condition, hydrogen atoms can be adsorbed with oxygen atoms, configuring the hydroxy group (O‐H) (Figure S4, Supporting Information). Moreover, the nano‐grain distribution has been statistically analyzed with RF plasma power variation. As the higher photo‐induced force corresponds to the grain boundary of nano‐grain (red area), lower photo‐induced force designates the vdW lattice (blue) (Figure 2e). Statistically extracting the nano‐grain distribution, nano‐grain area distributed as 107.29 nm^2^ (300 W), 179.96 nm^2^ (350 W), and 752.12 nm^2^ (400 W). Also, nano‐grain length has been extracted as 16.09 nm (300 W), 18.54 nm (350 W), and 34.57 nm (400 W) (Figure 5).

### 2.3 Evaluation of Bipolar Resistive Switching Performance

**Figure 3:**
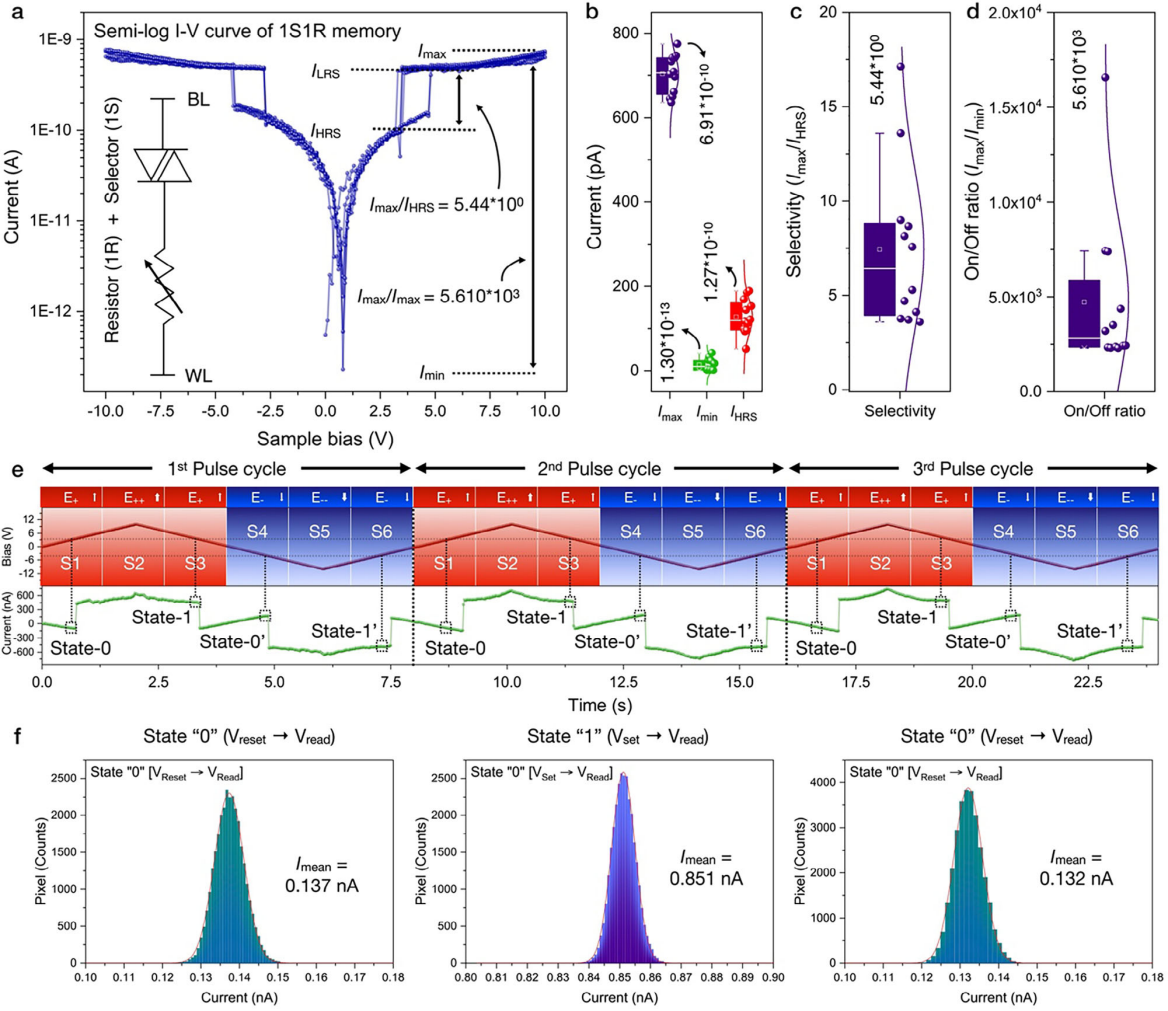
Bipolar resistive switching in vdW 2D/3D heterostructure. a) *I*–*V* curve measurements (3 cycles) of vdW 2D/3D heterostructure. A double hysteresis window has been observed at S1–S3 and S4–S6, which combines the resisitve memory (bulk vdW layers) and selctor (nano‐crystallized vdW layers). The clockwise *I*–*V* loop indicates bipolar resistive switching, which is governed by filament formation and rupture, modulated by the selector‐enabled current compliance. And its corresponding b) *I*_min_, *I*_max_, *I*_HRS_, c) selectivity (*I*_LRS_ / *I*_HRS_), d) on/off ratio (*I*_max_ /*I*_min_). e) Time‐resolved current mapping with pulse operation (3 cycles), generating the bipoalr resistive switching. f) Statical evalutation of HRS/LRS state with V_reset_ (‐2.5 V), V_set_ (+7.5 V) and V_read_ (+4.0 V), corresponding to the 0.137 nA (HRS [state “0”], V_reset_ (−2.5 V)→V_read_ (+4.0 V)), 0.851 nA (LRS [state “1”], V_set_ (+7.5 V)→V_read_ (+4.0 V)), and 0.132 nA (HRS [state “0”], V_reset_ (−2.5 V)→V_read_ (+4.0 V)).

To evaluate the performance of resistive memory, cyclic *I*–*V* curve has been measured (**Figure** **3**a). Regarding the vdW 2D/3D heterostructures, nano‐crystallized vdW lattice corresponds to the “resistor”, while the bulk vdW lattice operated as “selector”. Thus, the monolithically‐integrated nano‐crystallized VSe_2_ /bulk VSe_2_ heterostructure derives the 1S1R cell characteristics. Combining the selector (nano‐crystallized VSe_2_) and resistor (bulk VSe_2_), memory state can be activated with threshold voltage *V*_th_, which corresponds to the selector device operation. While the *I*_max_, *I*_min_, and *I*_HRS_ have been measured as 6.91 × 10^−10^ A, 1.30 × 10^−13^ A, and 1.27 × 10^−10^ A (Figure 3b). Based on these current values, selectivity (*I*_max_/*I*_HRS_) and on/off ratio (*I*_max_/*I*_min_) can be calculated as 5.44 × 10^0^ and 5.61 × 10^3^ (Figure 3c,d). Both in positive bias and negative bias range, the typical hysteresis loop has been symmetrically observed with bipolar switching as state [0, S1], state [1, S3], state [0′, S4], and state [1′, S6]. HRS switched to the LRS within a positive electric field (S1, *E*↑) at threshold voltage (*V*_th, S1_) of +4 .80 V. LRS can be switched back to HRS by decreasing the positive electric field (S3, *E*↑) at *V*_th, S3_ of +3.20 V, resulting the 1.6 V of hysteresis window. Reversely, LRS was switched to HRS with in negative electric field (S4, *E*↓) at *V*_th, S4_ of −4.10 V, and then switched back to LRS by decreased negative electric field (S6, *E*↓) within *V*_th, S6_ as −2.70 V, exhibiting symmetrical hysteresis window (1.60 V). Moreover, time‐resolved current can be analyzed within sequential bias pulse configuration (S1–S6) (Figure 3e). Selecting the bias value between the *V*_th, S1_ – *V*_th, S3_ and *V*_th, S4_ – *V*_th, S6_, current poses bipolar states (HRS [state “0”]/ LRS [state “1”]) for each bias value. Bipolar switching behavior are derived with possible combination of LRS /HRS alignment with electrical field, which is configured with *E*↑, HRS (state [0, S1]), *E*↓*P*↑ (state [0′, S4]), *E*↑, LRS (state [1, S3]) and *E*↓, HRS (state [1′, S6]). Also, statical evalutation of HRS/LRS has been conducted in Figure 3f. HRS/LRS state has been with switched with V_reset_ (‐2.5 V), V_set_ (+7.5 V) and V_read_ (+4.0 V). Current value statically measured as 0.137 nA in HRS [state “0”] (V_reset_ (‐2.5 V)→V_read_ (+4.0 V)), 0.851 nA in LRS [state “1”] (V_set_ (+7.5 V) →V_read_ (+4.0 V)), and 0.132 nA in HRS [state “0”] (V_reset_ (‐2.5 V)→V_read_ (+4.0 V)). Thus, the practical 1S1R application has been comprehensively validated within vdW 2D/3D heterostructures, which can be utilized as veraatile advances for M3D integration and its corresponding vdW device applications.

### 2.4 Spatial‐Resolved Resistive Switching Mechanism via C‐AFM Measurements

**Figure 4:**
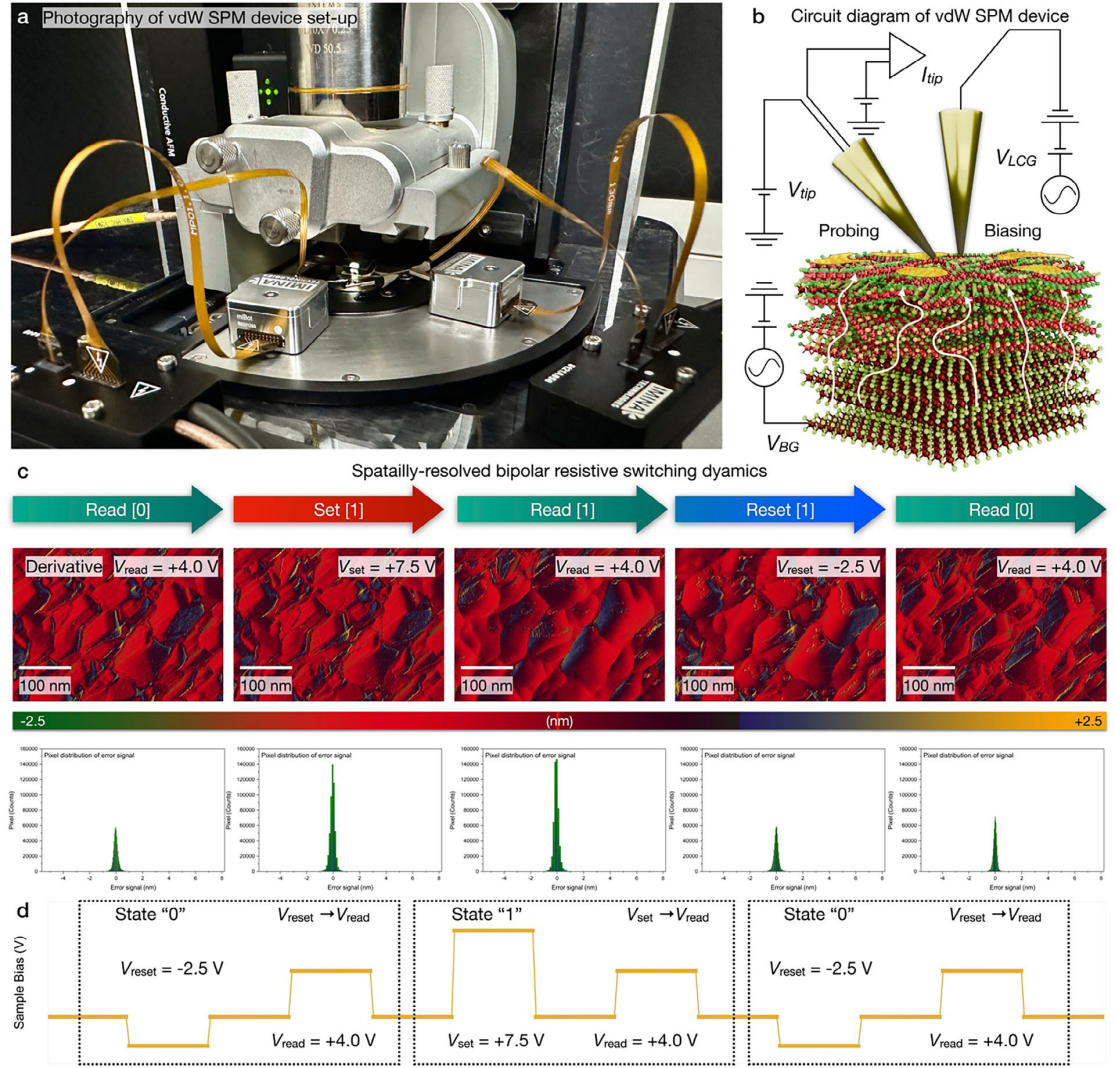
Spatially‐resolved resistive switching dynamics via conductive atomic force microscopy. a) Photography and b) schematic illustration of vdW SPM devive setup, configured with biasing tip and probing tip. Bias‐dependent topography image of nano‐crystallized vdW lattice, resulting in the conductive filaments activation. C‐AFM scan has been conducted with sequential “read” and “write” operation ([1] V_reset_ → [2] V_read_ [State “0”] → [3] V_set_ → [4] V_read_ [State “1”] → [5] V_reset_ → [6] V_read_ [State “0”]) c) Spatially‐resolved topography and its pixel distribution directly corresponds to the activation of conductive filaments and vertical ionic migration. As current image with V_read_ (+4.0 V) indicates the HRS [state “0”], LRS [state “1”] has been observed with (d) V_set_ (+7.5 V). After V_set_ (+7.5 V) scanning, LRS [state “0”] retained with V_read_ (+4.0 V) scan, indicating the non‐volatile memory operation.

As depicted in **Figure** **4**a,b, the C‐AFM system has been combined with a dual‐tip architecture with independently controlled biasing and probing electrodes. By decoupling voltage application from current detection, this configuration enables real‐time, spatially and temporally resolved imaging of resistive‐switching dynamics at the nanometer scale. As shown in Figure 4c, bias‐dependent topography image and pixel distribution has been spatially resolved with C‐AFM (Figures S6 and S7, Supporting Information), resulting in the local conductive filaments activation. C‐AFM scan has been conducted with sequential “read” and “write” operation, which has been constructed as [1] V_reset_ (−2.5 V)→ [2] V_read_ (+4.0 V) [Reading “0”] → [3] V_set_ (+7.5 V) → [4] V_read_ (+4.0 V) [Reading “1”] → [5] V_reset_ (−2.5 V)→ [6] V_read_ (+4.0 V) [Reading “0”]) (Figure S9, Supporting Information). Furthermore, sample DC bias was sequentially configured with positive “set” bias and negative “reset” bias to exclude the possibility of gradual dissipation of residual conductive filaments. Within “set” state, conductive filaments have been activated with positive bias, resulting in the topographical variation and LRS. After scanning with “set” state, “reset” scanning has been conducted with −2.5 V bias. As “reset” scan image exhibits the absence of topographical variation and conductance, blocking the activation of conductive filaments. When “set” bias has been sequentially increased with “reset” scan, topographical variation and conductance correlates with the sample DC bias, retaining the spatial reproducibility of conductive filaments. Within such bias configuration, spatially‐resolved topography and derivative image directly corresponds to the topography expansion, activation of conductive filaments, and vertical ionic migration (Figure S8, Supporting Information). To further validate the spatially‐resolved resistive switching dynamics, two‐box switching method has been applied. First, pre‐scan has been conducted with V_read_ (+4.0 V), which indicates the HRS [state “0”]. After V_read_ (+4.0 V) scan, LRS [state “1”] has been observed with V_set_ (+7.5 V) scan. As this resistive memory device operates as non‐volatile memory, LRS [state “1”] has been consistently obtained with V_set_ (+7.5 V). So, LRS/HRS has been concurrently observed with C‐AFM two‐box switching, which directly corresponds the bipolar resistive switching dynamics (Figure S9, Supporting Information).

### 2.5 Monolithic Control of 1S1R Memory Cell Device Function

**Figure 5:**
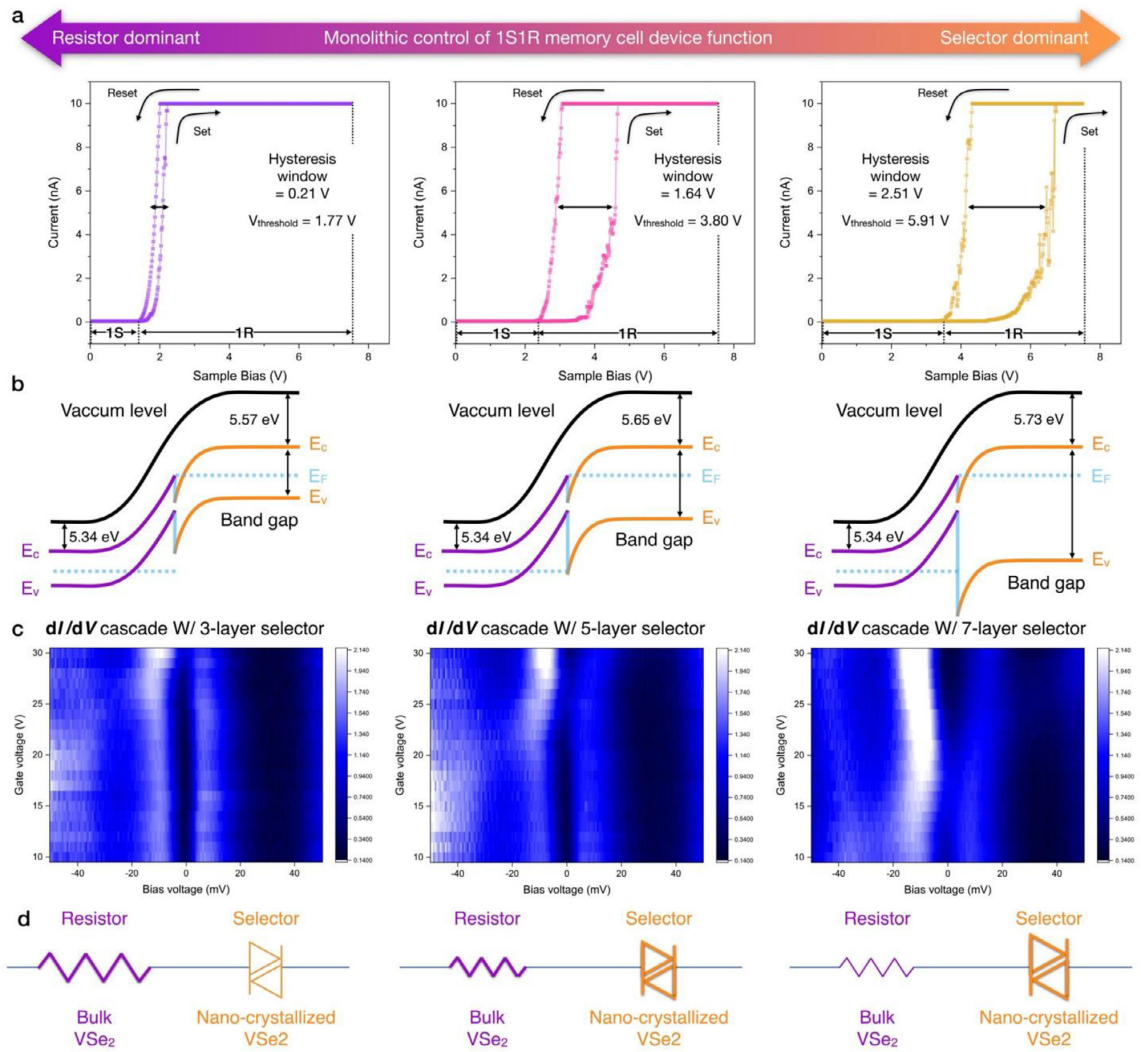
Monolithic control of 1S1R memory cell device function. a) Evaluation of hysteresis characteristics in 1S1R memory cell. Modulating the 1S (selector)/1R (resistive memory) ratio, the corresponding hysteresis window has been measured as 0.21 V, 1.64 V, and 2.51 V. b) Band alignment of monolithically‐integrated vdW 2D/3D heterostructure. c) Gate‐tunable d*I*/d*V* cascade mapping with selector layer control (3, 5, and 7 layers). A pronounced DOS suppression region (electronic bandgap) is observed near zero bias in all cases, with the width of this gap systematically increasing with selector dominance and gate bias modulation. d) Circuit symbol of 1S1R memory, indicating the dominant element of each 1S (selector)/1R (resistive memory) ratio.

To enable the practical implementation of monolithically integrated 1S1R resistive memory architectures, the vertical ratio between the 1S and 1R layers was precisely engineered through monolithic integration, as illustrated in **Figure** **5**a. By modulating the RF plasma power, the thickness ratio of the 1S/1R stack was systematically controlled, resulting in corresponding variations in the hysteresis window of the 1S1R device: 0.21 V for 3 selector layers, 1.64 V for 5 layers, and 2.51 V for 7 layers, respectively (Figure 5b). To achieve an artificial 2D/3D heterostructure, an Ar + H_2_S plasma treatment has been applied, where the plasma power was used to selectively induce nano‐crystallization in the top bulk vdW layers. Main key advantage of our top‐down approach is the ability to precisely control the number of sulfurized layers by tuning the RF plasma power. This allows artificial manipulation of both material properties and device characteristics. In contrast, bottom‐up methods such as epitaxial growth require high temperatures, template substrate, and typically suffer from low yield. Moreover, they lack precise control over the number of nano‐crystallized layers, making them unsuitable for the fabrication of integrated 1S1R cells where such control is critical. In comparison, our top‐down bulk vdW nano‐crystallization approach can be performed at room temperature and ensures uniform plasma treatment across the substrate. By seamlessly forming heterogeneous 2D vdW layers on pre‐existing 3D bulk vdW materials, artificial vdW 2D/3D heterostructure has been extensively fabricated and modulated. By varying the RF plasma power, the vertical thickness ratio between the 1S and 1R layers is finely tuned, enabling the transition between selector‐dominant and resistor‐dominant device behaviors. The resulting nano‐crystalline VSe_2_ seamlessly integrates with the underlying bulk lattice and functions as an ovonic threshold switch (OTS). When the applied voltage exceeds V_th_, a conductive path forms within the nano‐crystalline VSe_2_ via carrier injection and field‐induced trap filling, resulting in an abrupt transition from HRS to the LRS. Thus, the in‐situ integration of nano‐crystalline VSe_2_ OTS on a bulk vdW‐based ReRAM layer enables the realization of a monolithically fabricated 1S1R cell. To probe the electronic band structure of nano‐crystallized VSe_2_/bulk VSe_2_ heterostructure, gate‐dependent differential conductance (d*I*/d*V*) spectroscopy has been investigated with 3‐, 5‐, and 7‐layer selectors (Figure 5c). This systematic bandgap enlargement corresponds to the interlayer hybridization within the distorted van der Waals lattice. While the overall conductance increases with gate voltage (consistent with electrostatic doping), the bandgap position remains relatively stable. Furthermore, minor asymmetries in the d*I*/d*V* spectra directly reflect the asymmetrical band bending owing to the interfacial potential gradients. Such band gap variation of nano‐crystallized VSe_2_, which can be associated with enhanced ion migration and filament formation. As a nano‐crystallized lattice indicates an enhanced band gap in a laterally distorted domain, such nano‐crystallization induces conductive filaments to migrate through the grain boundary. While bulk VSe_2_ lattice generates conductive filaments with the electroforming process, resulting in a heterogeneous threshold voltage configuration (Figure 5d).

### 2.6 Synaptic Response of Monolithically‐Integrated 1S1R Memory Cell Device

**Figure 6:**
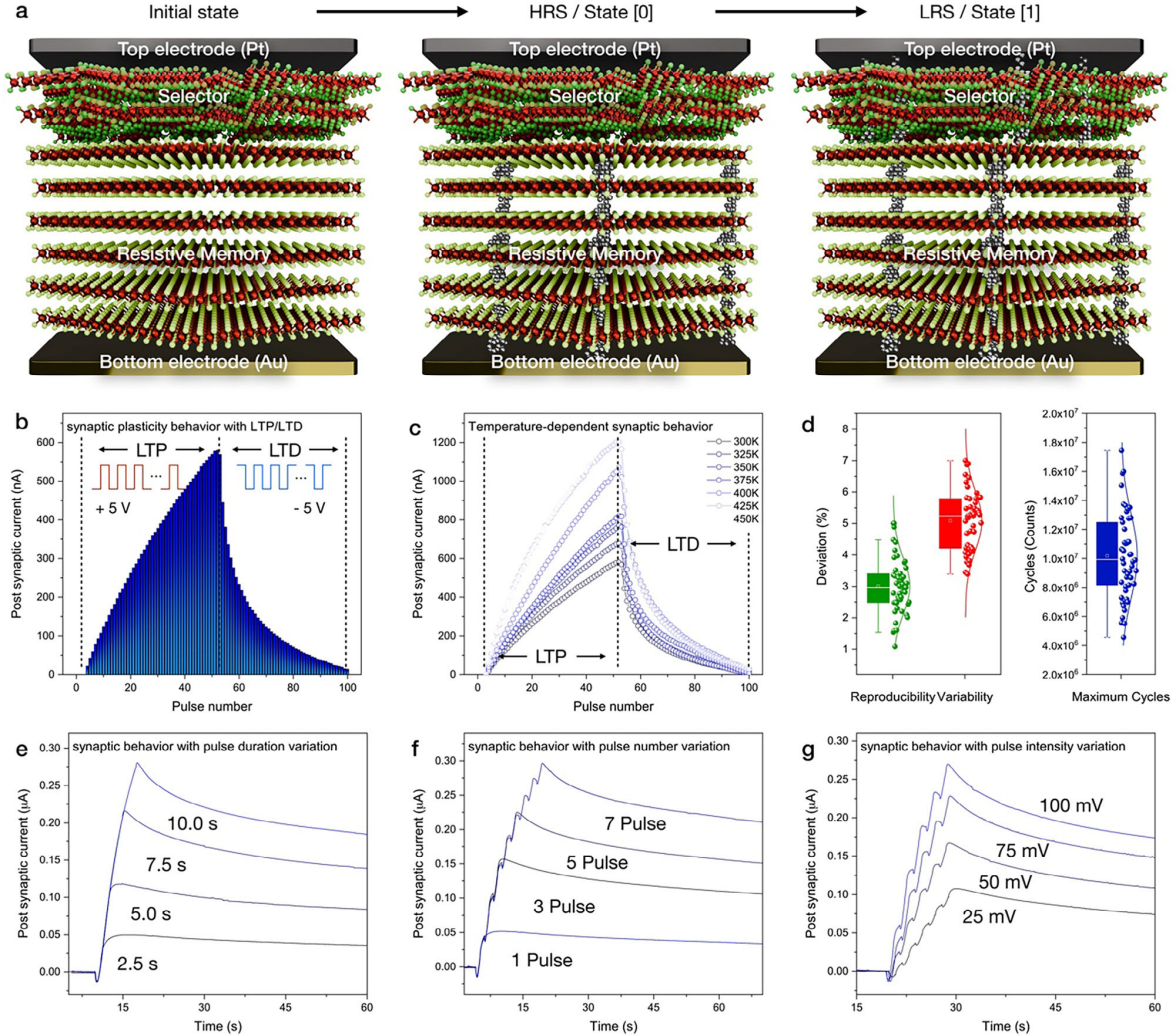
Synaptic response of monolithically‐integrated vdW 1S1R memory function. a) Schematic illustration of the resistive switching mechanism in vdW 2D/3D heterostructure, separating the HRS and LRS with a heterogeneous bandgap. b) Synaptic plasticity with LTP/LTD (±5 V Pulse, 100 pulses), and its c) temperature‐dependent synaptic plasticity with 300–450 K variation (±5 V Pulse, 100 pulses). d) Statistical evaluation of device‐to‐device reproducibility, device‐to‐device variation, and endurance (maximum cycles) of 50 devices. e) Synaptic behavior with heterogeneous pulse durations (2.5, 5.0, 7.5, and 10.0 s), pulse numbers (1, 3, 5, and 7), and pulse intensities (25, 50, 75, and 100 mV).

While bulk VSe_2_ lattice generates conductive filaments with the electroforming process, resulting in a heterogeneous threshold configuration. Cross‐sectional schematic of the HRS and LRS indicates the resistive switching mechanism based on ion‐assisted grain boundary modulation, filament, ion dynamics, and grain boundary‐induced conduction (**Figure** **6**a). Robust long‐term potentiation and depression (LTP/LTD) characteristics have been demonstrated in our monolithically‐integrated 1S1R device under repeated pulse stimulation, as shown in Figure 6b. Temperature‑dependent measurements from 300K to 450K demonstrate LTP/LTD behavior up to 450K (Figure 6c). The linear increase in post‑synaptic current with temperature reflects enhanced ionic mobility within the nano‑crystalline VSe_2_ selector, yet the absence of abrupt conductance failures occurs, indicating the thermal robustness of the vdW 2D/3D heterostructure for stable neuromorphic operation. All devices exhibit a clockwise *I*–*V* hysteresis loop, indicative of bipolar switching governed by field‑induced filament growth and rupture stabilized by the nonlinear threshold behavior. For over 50 cells, the standard deviation of switching voltages and current levels remains below 5% (Figure 6d), confirming the reproducible compliance control provided by the integrated selector. Under ambient conditions, the monolithic 1S1R cells sustain >1.8 × 10⁷ switching cycles (Figure 6d). The device exhibited stable endurance characteristics up to ≈10^7^ switching cycles at both 300 K and 450 K, with negligible degradation in HRS/LRS values. The consistent resistance states across the entire cycling range confirm the robust operational stability of the monolithically integrated architecture (Figure S10, Supporting Information). Modulating the electrical pulse parameters (number, duration, intensity), the 1S1R cells exhibit a gradual conductance variation of analog synapses (Figure 6e–g). Owing to the nano‑crystallization, decreased conductance of selector layer limits abrupt current increment, enabling linear weight updates across multiple resistance states with minimal nonlinearity. Previously, synaptic weight updates have been reported at the interfaces of vdW heterostructures,^[^ ^36^ ^]^ such synaptic behavior biologically mimics weight adjustment and demonstrates neuromorphic plasticity through controllable LTP/LTD pulse characteristics. Similarly, our artificial vdW 2D/3D heterostructure (nano‐crystalline VSe_2_/bulk VSe_2_ interface) enables the synaptic functionality with interfacial charge accumulation at the vdW interface. C‐AFM measurements revealed that increasing either the contact area (400, 1225, and 2500 nm^2^) or the contact force (50, 75, and 100 nN) led to the slight increase in LRS current (Figure S10, Supporting Information). This increment can be attributed to a reduced contact resistance arising from an enlarged device contact area, as well as an increased effective conductive cross‐section or number of parallel conductive filaments within the active layer. Furthermore, enhanced local pressure and associated Joule heating further promote defect generation and migration, facilitating filament growth and consequently lowering LRS resistance. These synergistic effects enable the area‐ and force‐dependent LRS modulation behavior.

## 3 Conclusion

In summary, a monolithically‐integrated 1S1R cell has been presented with vdW 2D/3D heterostructure, which addresses longstanding challenges in 3D vertical device integration technologies. The synergistic incorporation of Ar + H_2_S plasma sulfurization induces nano‐grain formation at the bulk VSe_2_, yielding stable bipolar resistive switching behavior. The monolithic‐integrated 1S1R cell exhibits *I*_max_, *I*_min_, and *I*_HRS_ as 6.91 × 10^−10^ A, 1.30 × 10^−13^ A, 1.27 × 10^−10^ A, resulting in the low leakage currents, robust switching ratios, and reliable bipolar memory states. As bipolar switching mechanism has been sequentially resolved with “read” and “write” operation, LRS/HRS has been concurrently observed with two‐box switching, which directly corresponds the bipolar resistive switching dynamics. To achieve the practical application of monolithically‐integrated resistive memory, the ratio of 1S (selector)/1R (resistive memory) has been monolithically controlled. Moreover, synaptic behavior has been validated in monolithically‐integrated 1S1R device with repeated LTP/LTD pulse stimulation, resulting the gradual conductance variation of analog synapses, while sustaining the standard deviation of switching voltages and current levels remains as 5% and maximum switching cycles as 1.8 × 10⁷ switching. In conclusion, we envision that our monolithically‐integrated 1S1R cell can offer a generalizable platform for next‐generation 3D integrated neuromorphic device and AI hardware.

## 4 Experimental Section

### Mechanical Exfoliation and Transfer of vdW VSe_2_

Before mechanical exfoliation and dry transfer, a polydimethylsiloxane stamp was attached to a glass cover. vdW VSe_2_ were mechanically exfoliated from bulk crystals (HQ Graphene, Netherlands) onto polydimethylsiloxane stamps and then transferred onto the substrate by applying a transfer condition of 70 °C.

### vdW 2D/3D Heterostructures Fabrication for Monolithically‐Integrtated 1S1R Cell

In vdW 2D/3D heterostructures, the nano‐crystallized vdW lattice functions as the resistive element, while the bulk vdW lattice serves as the selector, enabling monolithically‐integrated 1S1R configurations. To induce nano‐crystallization of VSe_2_, inductively coupled plasma‐enhanced chemical vapor deposition (ICP‐PECVD, AFS‐IC6T, Korea) was employed. Prior to plasma treatment, the chamber was evacuated to a high vacuum (≈10^−5^ Torr) to eliminate contaminants and suppress undesired reactions during synthesis. The RF plasma power was maintained at 400 W, with constant gas flow conditions of Ar and H_2_S (50 sccm each) at 25 mTorr pressure and room temperature, ensuring fabrication of vdW 2D/3D heterostructures.

### AFM Measurements

AFM (NX‐10 AFM, Park Systems, Republic of Korea) measurements were conducted with an Electri‐Multi75G cantilever. A silver paste electrode (Elcoat P‐100, CANS, Japan) was selectively deposited on the sample edge to induce electrical contact. The ElectriMulti75‐G cantilever was calibrated with a tip radius of 25 nm, a length of 225 µm, a height of 17 µm, a width of 28 µm, and a spring constant of 3.3 N m^−1^, resulting in a resonance frequency of 60.8 kHz. C‐AFM measurements with a continuous voltage waveform, which was configured with 8 s for one cycle (3 cycle measurements), and sample bias range as −10 –+10 V (V_max_ as +10 V/ V_min_ as −10 V). Current‐voltage characteristics through the sulfurized vdW materials were probed within the CAFM probe (Electri‐Multi75G, tip radius as 25 nm) to operate as the top electrode of the vdW materials, grounded to the Au substrate. In addition, PiFM (NX‐IR, Park Systems, Republic of Korea) with a PPP‐NCHR cantilever was employed for the PiFM measurements. The QCL laser used for PiFM was adjusted to an intensity of 1%. Prior to the PiFM measurements, the QCL laser was focused on the initial spatial intensity positions directly under the AFM tip. Customized vdW SPM device set‐up was installed with Mibot (IMINA, Switzerland) and AFM (NX 10, Park Systems, South Korea) with Tungsten probe (PT‐010N‐15‐B8) with 10 nm tip radius, 15 mm in length), enabling the electrical pulse measurements and *I*–*V* curve sweep (Bias range −170 V–170 V, 60 Hz, AC ±107 V).

### Material Characterization

XPS measurements (XIS Supra+, Kratos, United Kingdom) were used to characterize vdW materials, with an X‐ray spot size of 400 µm. Peak deconvolution was performed with the profiles aligned using the C 1s peak at 285 eV. The XPS data were calibrated using the CASAXPS software (version 8.1). Optical microscopy (U‐MSSP4, Olympus, Japan) and FE‐SEM (S‐4800, Hitachi, Japan) were used to examine the transferred flakes. For cross‐sectional TEM specimen preparation, a focused ion beam instrument (NX2000, Hitachi Ltd., Japan) was used, employing a Ga^+^ ion beam (30–5 keV) and a lift‐off process to etch the specimens. TEM (JEM‐2100F, JEOL, Japan) and XRD (Empyrean, Malvern PANalytical, United Kingdom) were used to observe the lattice structure, EDS, and SAED patterns of the sulfurized VSe_2_ structures at the atomic scale.

## Conflict of Interest

The authors declare no conflict of interest.

## Author Contributions

J.L., G.K., H.S., and S.H. contributed equally to this work. J.L., G.K., H.S., and S.H. prepared samples and performed experiments. G.K., H.C., H.K., S.S., S.S., H.L., C.P., H.S., H.H., G.B., H.S., D.C., and D.L. performed the technical discussions on resistive switching mechanism. Y.C., G.H., G.J., and Y.L. provided technical advice on the PiFM systems. T.T. and K.W. provide the bulk hexagonal boron nitride samples. M.G. and A.B. include in Raman spectroscopy measurements. Y.K., S.K., and S.B. included in the technical discussions about artificial 2D/3D heterostructure. A.O., Y.K., and L.F., A.H., W.J., J.L., G.K., H.S., and S.H. wrote the manuscript with contributions from all the authors. T.K. designed and supervised the study. All the authors have read and approved the final version of this manuscript.

## Supporting information



Supporting Information

## Data Availability

The data that support the findings of this study are available from the corresponding author upon reasonable request.
