# Supplementary material for: Monolithically‐Integrated van der Waals Synaptic Memory via Bulk Nano‐Crystallization
Source: Adv Sci (Weinh). 2025 Aug 26;12(43):e10961. doi: 10.1002/advs.202510961 (PMC12631876; doi:10.1002/advs.202510961)
Supplement: Supplementary file 1 — Supporting Information [file ADVS-12-e10961-s001.docx]

Supporting Information

Monolithically-integrated van der Waals Synaptic Memory *via* Bulk Nano-crystallization

*Jinhyoung Lee^†^, Gunhyoung Kim ^†^, Hyunho Seok^†^, Sujeong Han ^†^, Yoonmi Cha, Sihoon Son, Hyunbin Choi, Magdalena Grzeszczyk, Aleksander Bogucki, Yunseok Choi, Seungil Kim, Hyeonjeong Lee, Chaerin Park, Geonwook Kim, Hosin Hwang, Hyunho Kim, Dongho Lee, Seowoo Son, Geumji Back, Hyelim Shin, Alexina Ollier, Yeon-Ji Kim, Lei Fang, Gyuho Han, Goo-Eun Jung, Youngi Lee, Hyeong-U Kim, Kenji Watanabe, Takashi Taniguchi, Sanghoon Bae, Andreas Heinrich, Won-Jun Jang, and Taesung Kim^*^*

J. Lee, H. Lee, G. Kim, D. Lee, Prof. T. Kim

School of Mechanical Engineering, Sungkyunkwan University (SKKU), Suwon-si, Gyeonggi-do 16419, South Korea

J. Lee, Y. Kim, Dr. A. Ollier, Prof. Dr. L. Fang, Prof. A. Heinrich, Dr. W. Jang

Center for Quantum Nanoscience, Institute for Basic Science (IBS), Seoul 03760, South Korea

S. Han, G. Kim, H. Choi, C. Park, H. Hwang, H. Kim, H. Shin, G. Back, Prof. T. Kim

Department of Semiconductor Convergence Engineering, Sungkyunkwan University, Suwon 16419, South Korea

S. Son, S. Son, Prof. T. Kim

SKKU Advanced Institute of Nanotechnology (SAINT), Sungkyunkwan University, Suwon 16419, South Korea

S. Son, S. Son, Prof. T. Kim

Department of Nano Science and Technology, Sungkyunkwan University, Suwon 16419, South Korea

Dr. H. Seok

Research Laboratory of Electronics, Massachusetts Institute of Technology, Cambridge, MA, 02139, USA

Y. Cha, G. Han, Dr. G, Jung, Y. Lee

Park Systems Corporation, 109, Gwanggyo-ro, Yeongtong-gu, Suwon-si, Gyeonggi-do, 16229, South Korea

Dr. Y. Choi, Dr. S. Kim, Prof. S. Bae

Department of Mechanical Engineering and Materials Science and Institute of Materials Science and Engineering, Washington University in St. Louis, MO, 63130, USA

Dr. H. Kim

Semiconductor Manufacturing Research Center, Korea Institute of Machinery and Materials (KIMM), Daejeon 34103, South Korea

Dr. H. Kim

Nano-Mechatronics, KIMM Campus, University of Science & Technology (UST), Daejeon 34113, South Korea

Dr. K. Watanabe, Dr. T. Taniguchi

National Institute for Materials Science, Namiki 1-1, Tsukuba, 305-0044, Ibaraki, Japan

Dr. M. Grzeszczyk, Dr. A. Bogucki, Y. Kim, Dr. A. Ollier, Dr. Lei Fang, Prof. A. Heinrich, Dr. W. Jang

Department of Physics, Ewha Womans University, Seoul 03760, South Korea

Prof. T. Kim

Department of Nano Engineering, Sungkyunkwan University, Suwon 16419, South Korea

*†* These authors contributed equally to this work.

^*^Corresponding Authors: [tkim@skku.edu](mailto:tkim@skku.edu)

**Keywords:** 2D/3D heterostructures, Synaptic memory, 1S1R cell, Resistive switching, Atomic force microscopy;

**
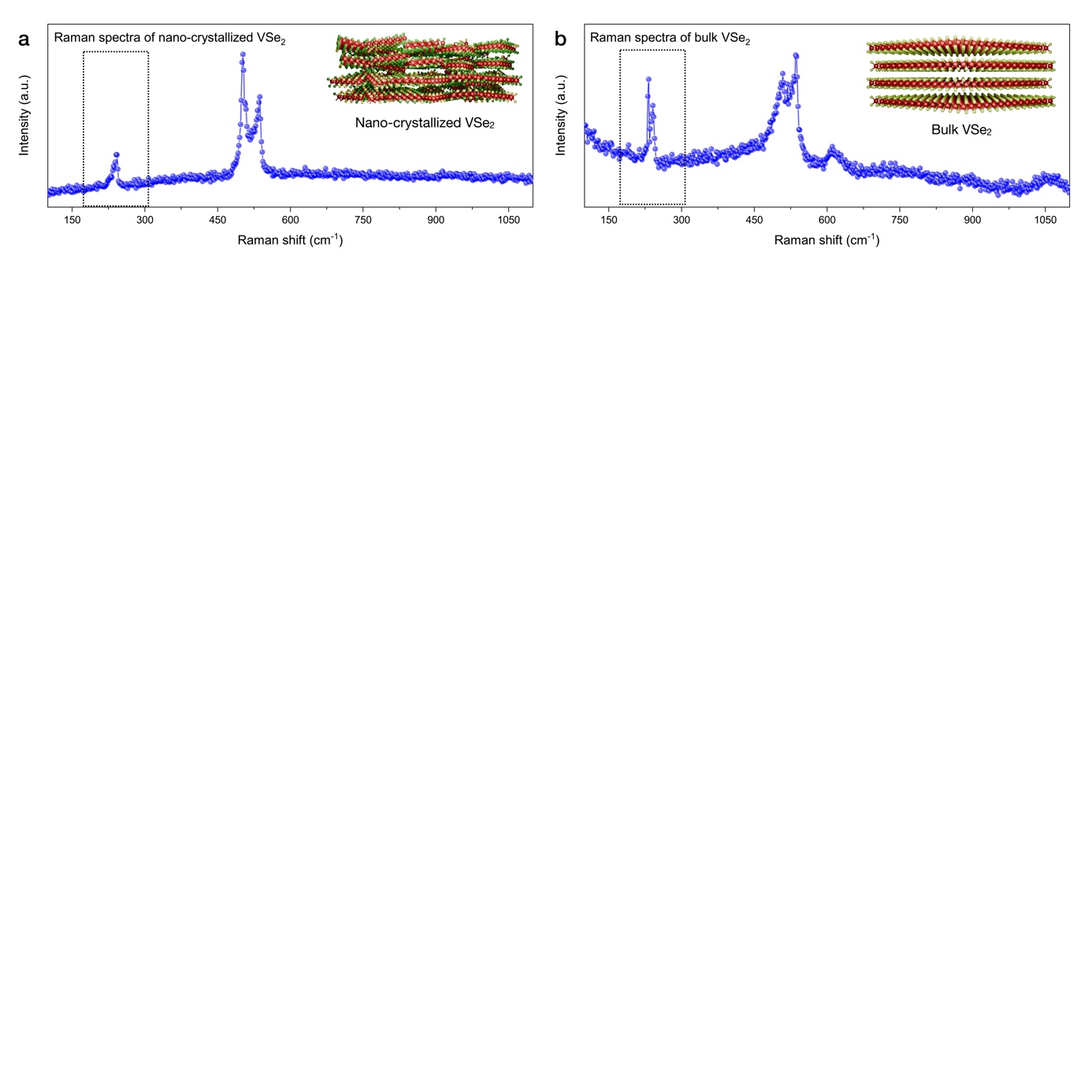
**

**Figure S1. Raman spectra of bulk VSe_2_ and nano-crystallized VSe_2_.** full Raman spectra of (a) bulk VSe_2_ and (b) nano-crystallized VSe_2_, indicating the decreased *A*_1g_ peak and increased *E*_2g_ peak.


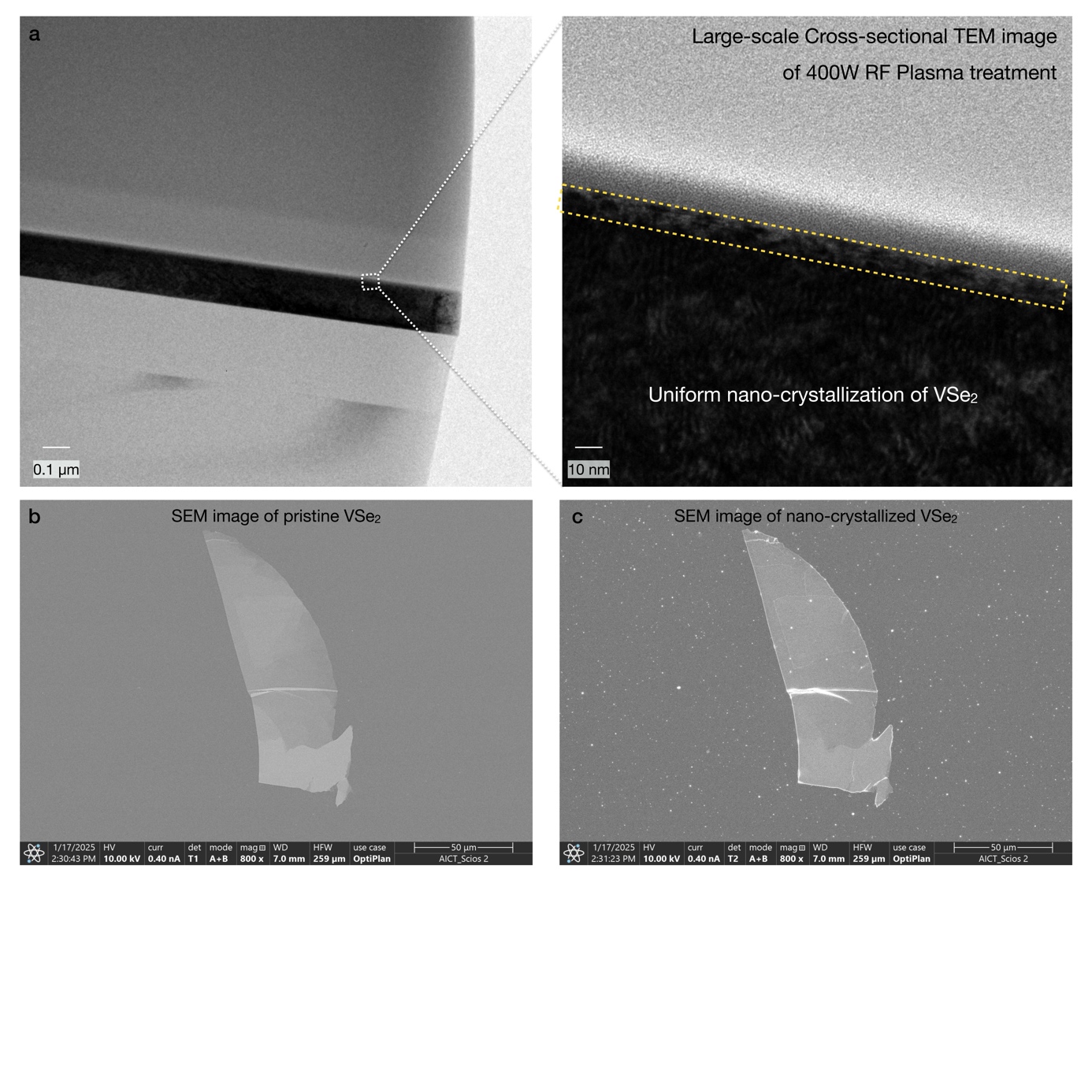


**Figure S2. SEM and Large-scale cross-sectional TEM image of 400 W RF sulfurization.** (a) Cross-sectional TEM image, SEM image of (b) bulk VSe_2_ and (c) nano-crystallized VSe_2_, exhibiting the ion bombardment effects.

*
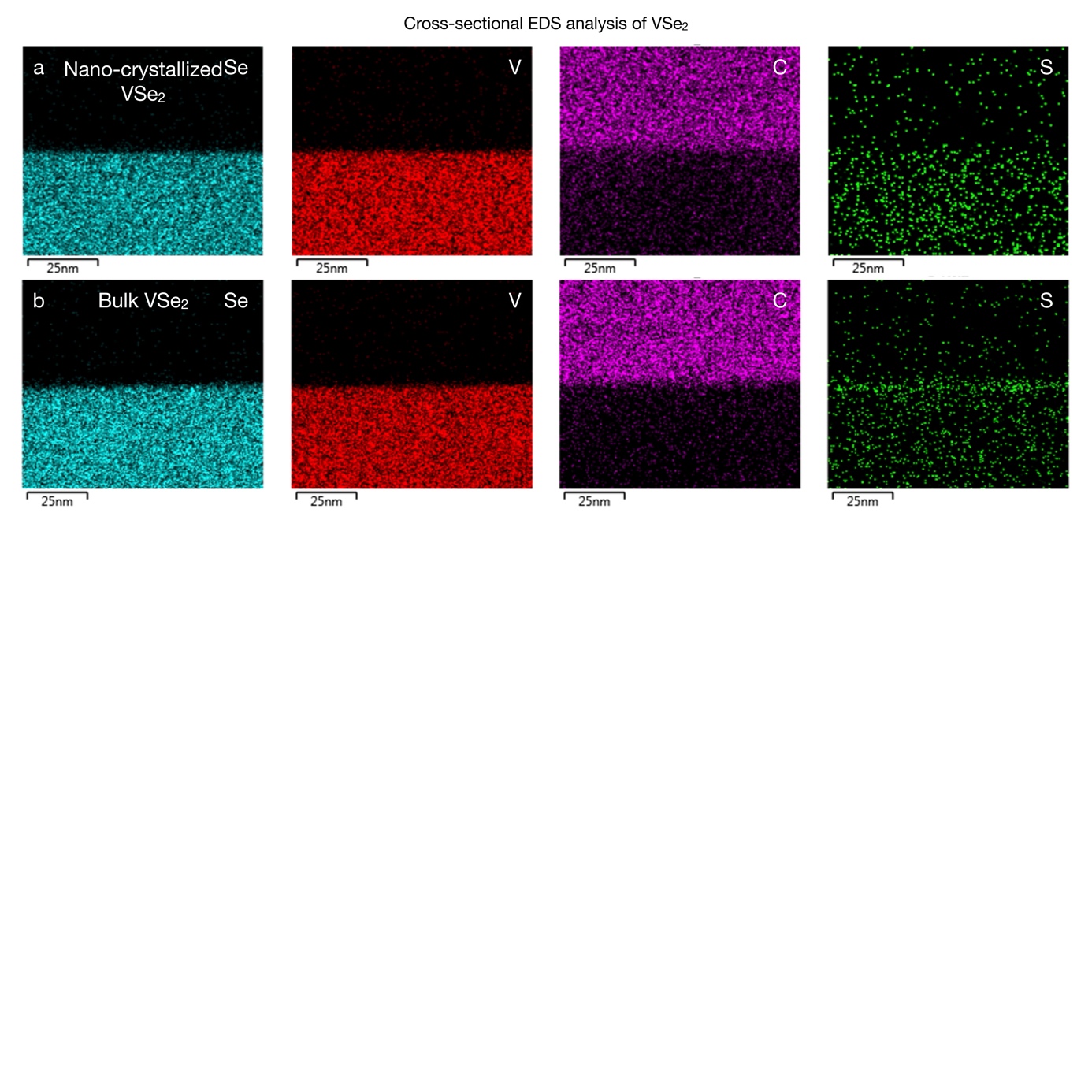
*

**Figure S3. Cross-sectional EDS mapping of bulk VSe_2_ and nano-crystallized VSe_2_.** Cross-sectional EDS mapping of (a) bulk VSe_2_ and (b) nano-crystallized VSe_2_, indicating the homogeneous V, Se, C atomic distribution and heterogeneous S atomic distribution.


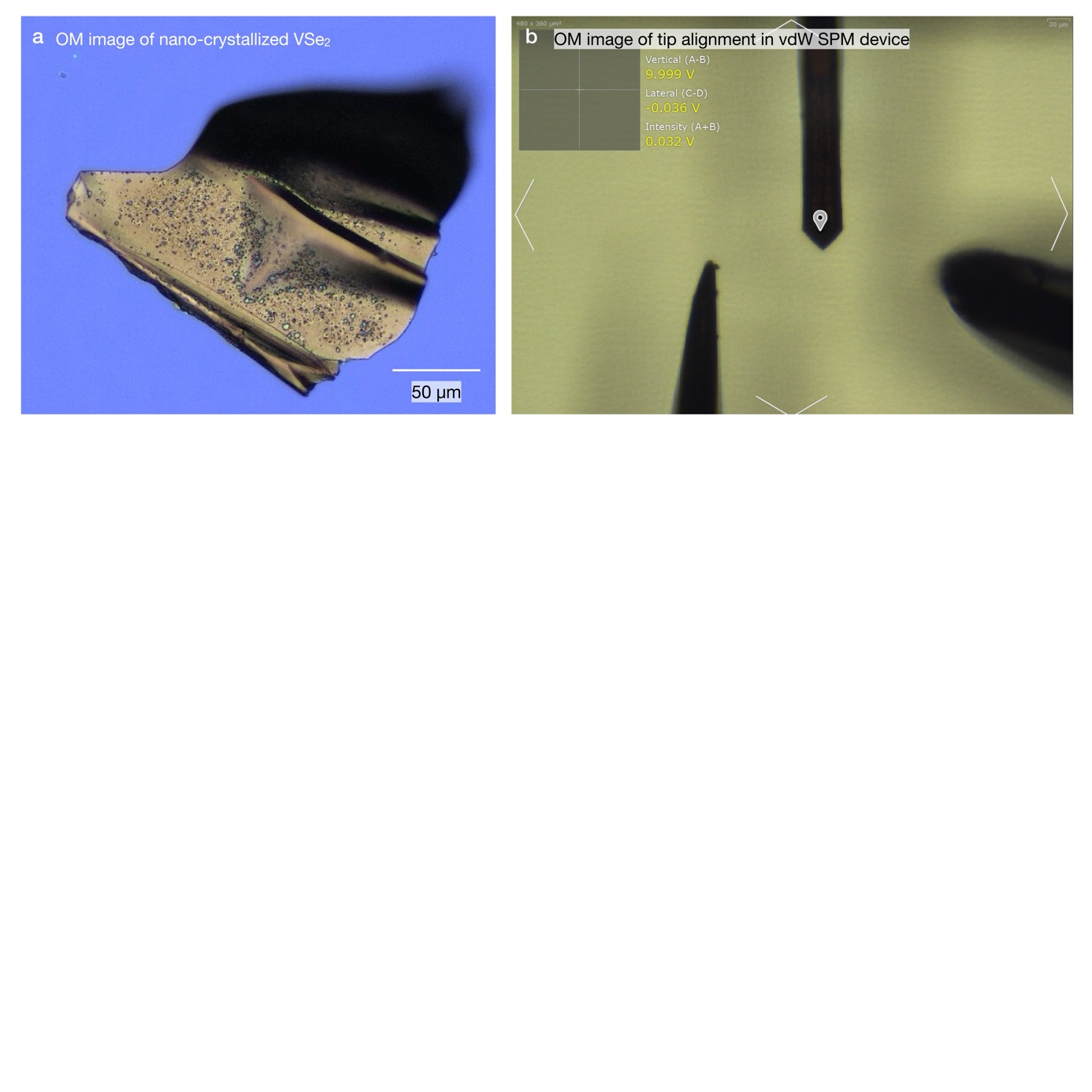


**Figure S4. OM image of nano-crystallized VSe_2_ and tip alignment**. (a) Optical microscopy image of nano-crystallized VSe₂ flake (b) tip alignment of vdW SPM device.


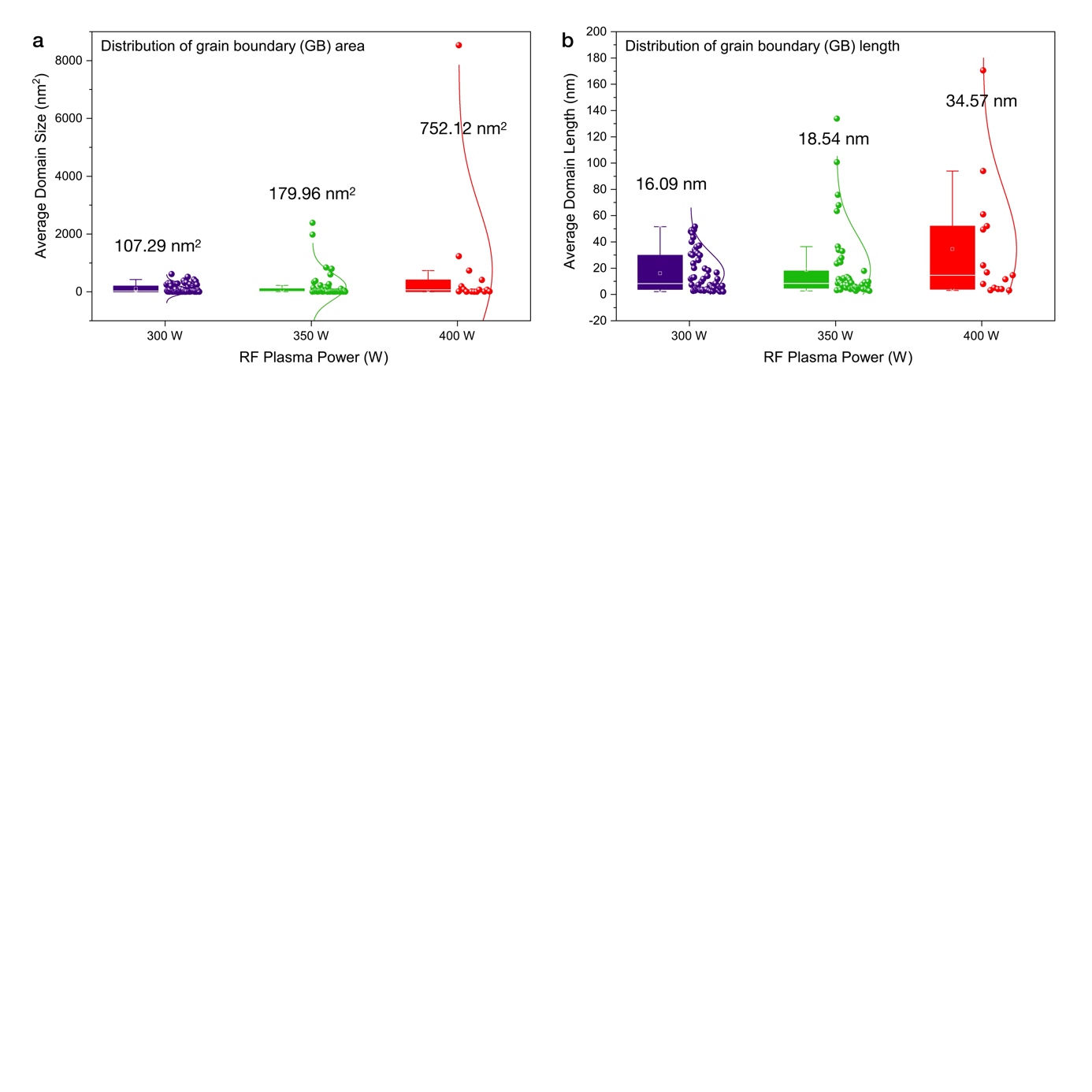


**Figure S5. Statistical distribution of grain boundary.** Statistical evaluation of a) grain boundary area and b) grain boundary length with RF power distribution.

**
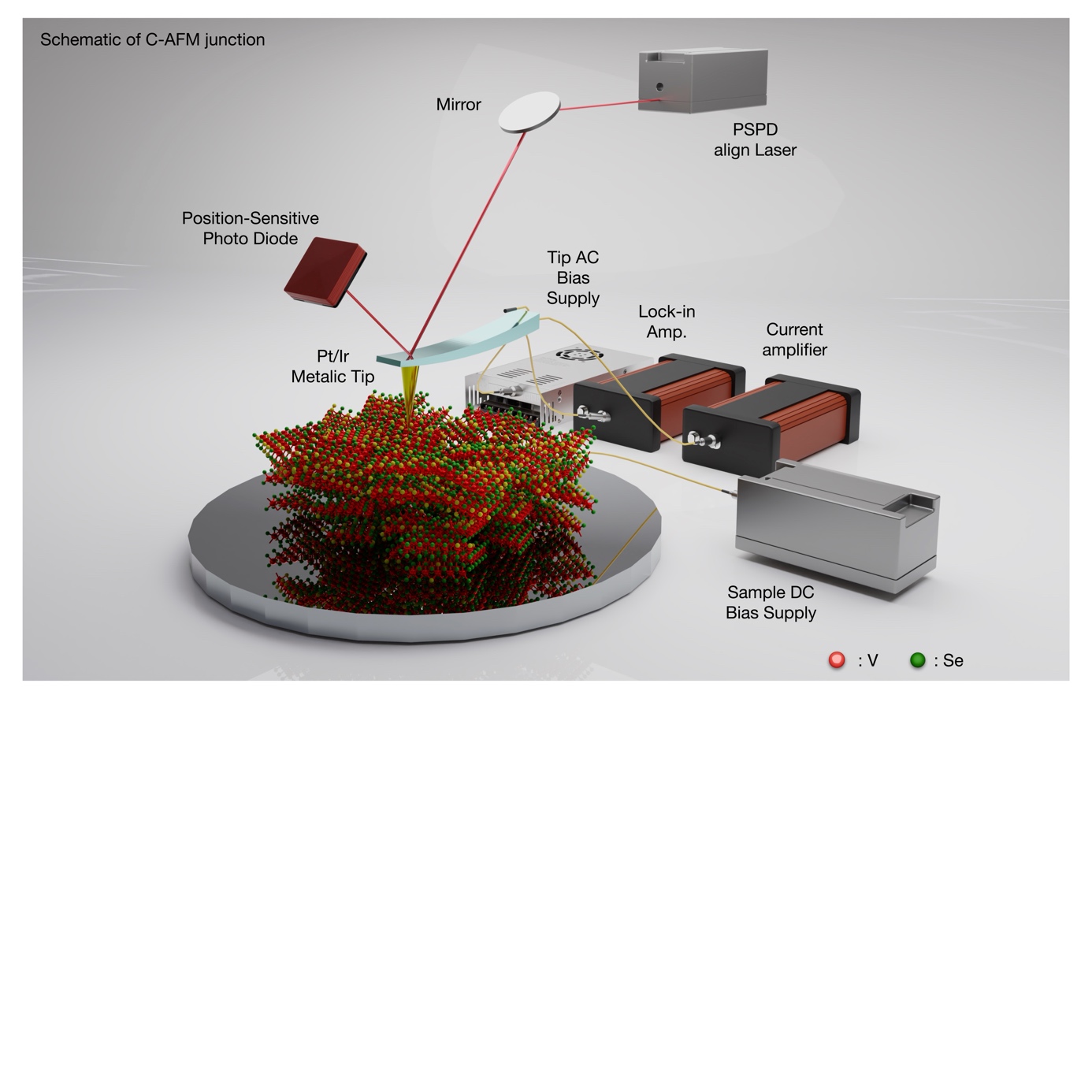
**

**Figure S6. Schematic illustration of C-AFM junction.** Illustration of C-AFM junction, consisting of Pt/Ir tip, position-sensitive photo diode (PSPD), lock-in amplifier, current amplifier, sample direct current (DC) bias, and tip DC bias.


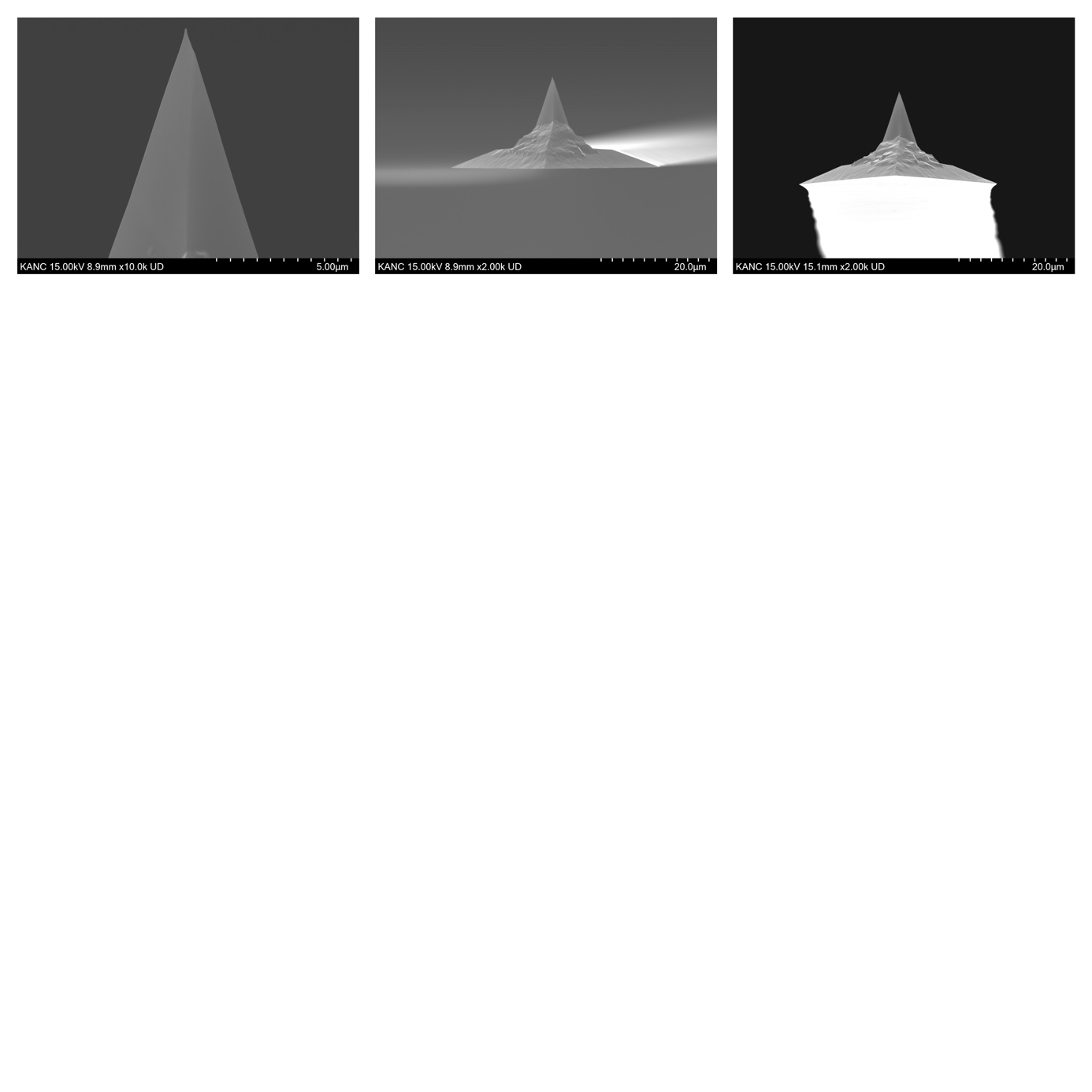
 **Figure S7. SEM image of C-AFM tip.** SEM image C-AFM tip and detail image of tip apex site (tip radius as 25 nm).


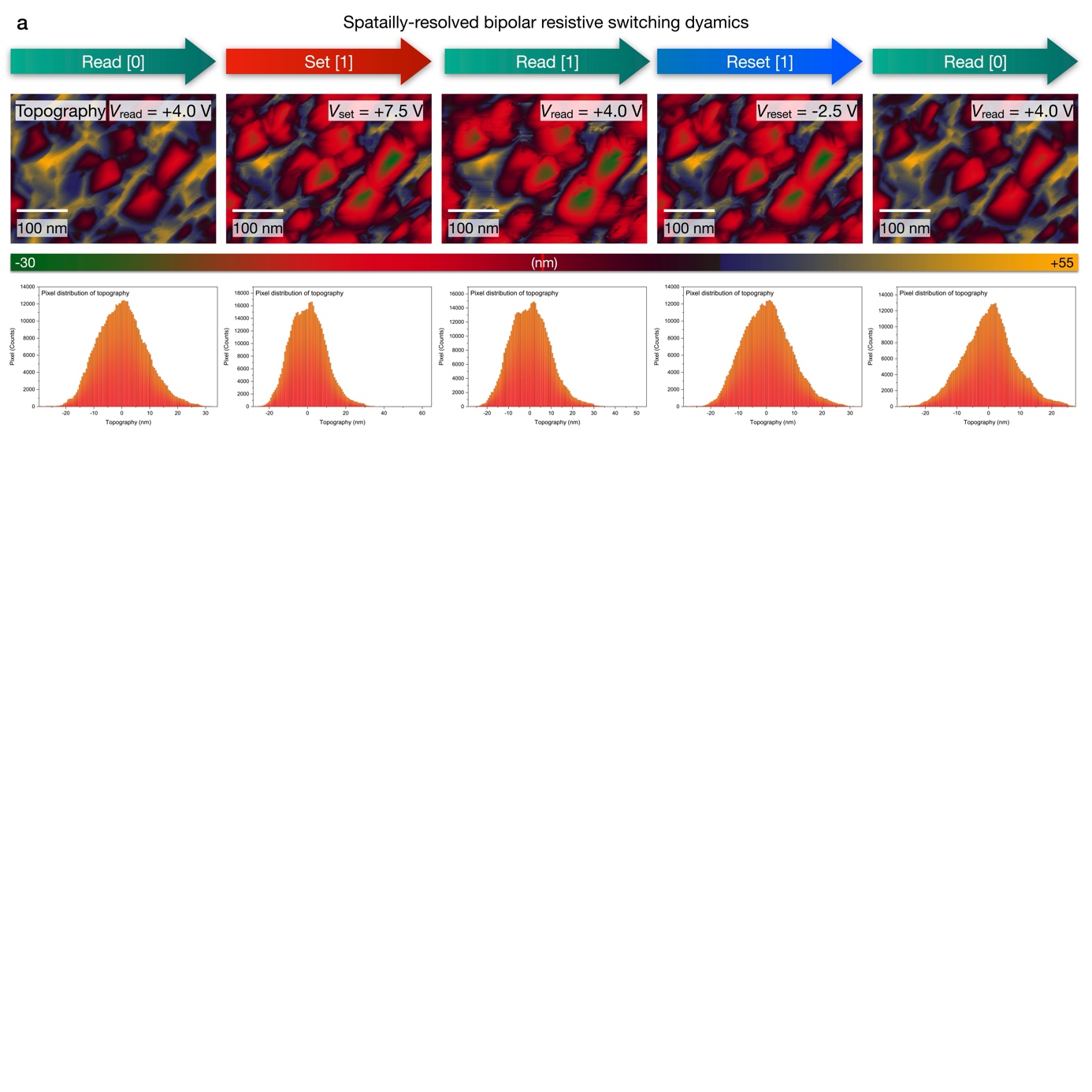


**Figure S8. Spatially-resolved bipolar resistive switching with derivative images of 3D topography.** Sequential topography imaging of nano-crystallized vdW lattice, resulting in the conductive filaments activation. C-AFM scan has been conducted with sequential “read” and “write” operation ([1] V_reset_ → [2] V_read_ [State “0”] → [3] V_set_ → [4] V_read_ [State “1”] → [5] V_reset_ → [6] V_read_ [State “0”]) Spatially-resolved derivative images and its pixel distribution directly corresponds to the activation of conductive filaments and vertical ionic migration.


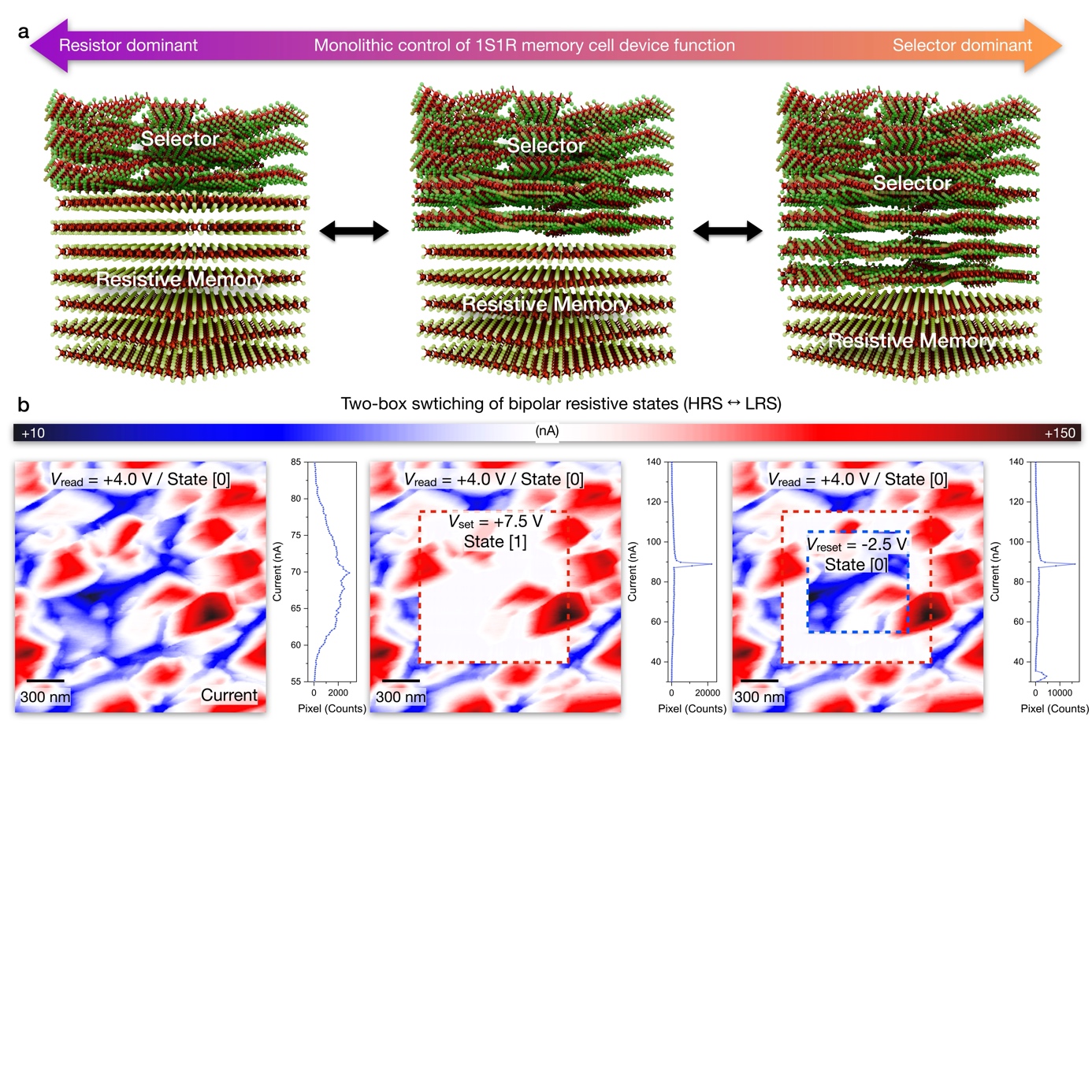


**Figure S9. Monolithic control of bulk nano-crystallization of VSe_2_ vdW layer**. (a) Schematic illustration of monolithic control of 1S (selector)/1R (resistive memory) ratio. (b) Two-box switching of bipolar resistive states. As current image with V_read_ (+4.0 V) indicates the HRS [state “0”], LRS [state “1”] has been observed with V_set_ (+7.5 V). After V_set_ (+7.5 V) scanning, LRS [state “0”] retained with V_read_ (+4.0 V) scan, indicating the non-volatile memory operation.


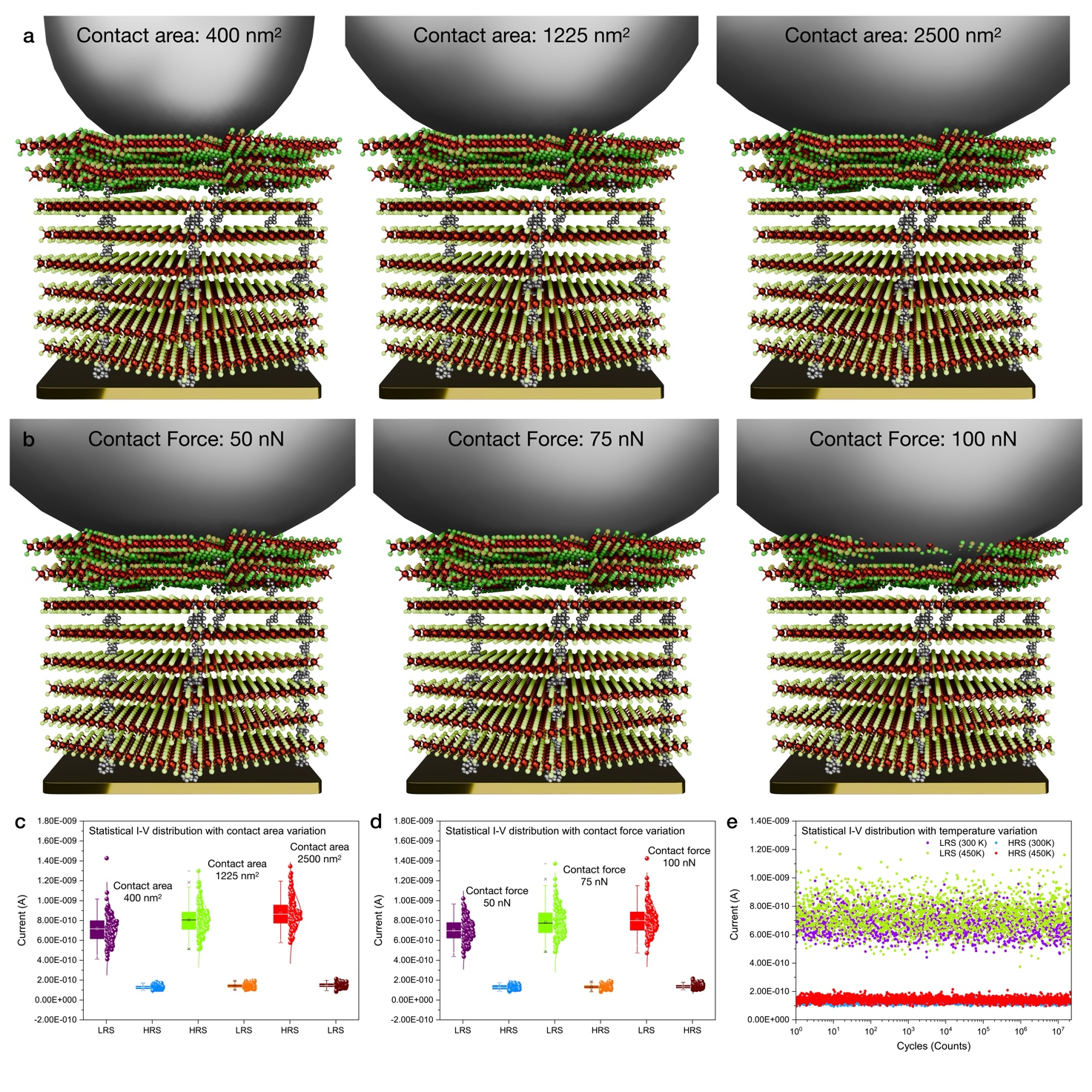


**Fig. S10| Contact engineering of nano-crystallized vdW lattice.** (a) Schematic illustration of contact area engineering with AFM tip radius. (b) Schematic illustration of contact force engineering with AFM tip setpoint. (c) Statistical HRS/LRS evaluation of (c) contact area variation (400 nm^2^, 1225 nm^2^, and 2500 nm^2^) (d) contact force variation (50 nN, 75 nN, and 100 nN), and (e) endurance test (~10^7^) with specific HRS/LRS values.
